# Competitive Interaction of *Axonopus compressus* and *Asystasia gangetica* under Contrasting Sunlight Intensity

**DOI:** 10.1155/2013/308646

**Published:** 2013-09-18

**Authors:** B. Samedani, A. S. Juraimi, M. P. Anwar, M. Y. Rafii, S. H. Sheikh Awadz, A. R. Anuar

**Affiliations:** ^1^Department of Crop Science, Faculty of Agriculture, Universiti Putra Malaysia, 43400 UPM, Serdang, Selangor, Malaysia; ^2^Institute of Tropical Agriculture, Universiti Putra Malaysia, 43400 UPM, Serdang, Selangor, Malaysia; ^3^Department of Land Management, Faculty of Agriculture, Universiti Putra Malaysia, 43400 UPM, Serdang, Selangor, Malaysia

## Abstract

*Axonopus compressus* is one of the native soft grass species in oil palm in Malaysia which can be used as a cover crop. The competitive ability of *A. compressus* to overcome *A. gangetica* was studied using multiple-density, multiple-proportion replacements series under a glasshouse and full sunlight conditions in a poly bag for 10 weeks. *A. compressus* produced more dry weight and leaf area when competing against *A. gangetica* than in monoculture at both densities in the full sunlight and at high density in the shade. Moreover, the relative yield and relative crowding coefficients also indicated *A. compressus* is a stronger competitor than *A. gangetica* at both densities in the full sunlight and high density in the shade. It seemed that *A. gangetica* plants in the shade did not compete with each other and were more competitive against *A. compressus* as could influence *A. compressus* height in the shade. It is concluded that although suppression of *A. gangetica* by *A. compressus* occurred under full sunlight, irrespective of plant density, this ability reduced under shade as *A. compressus* density decreased. The result suggests that *A. compressus* in high density could be considered as a candidate for cover crops under oil palm canopy.

## 1. Introduction

Oil palm is the number one cash crop in Southeast Asia, especially in Malaysia and Indonesia. Worldwide coverage of oil palm plantations is 13 million ha of which about 5.3 million ha lies in Indonesia and 4.2 million ha in Malaysia [1]. The high demand for vegetable oil has led to the expansion of the area covered by oil palm plantations in this region.

In immature oil palm plantations, vacant space between palms creates opportunities for noxious weeds to grow ubiquitously. Noxious weeds such as *Chromolaena odorata*, *Mikania cordata,* and *Mikania micrantha* compete with the oil palm for nutrients, moisture, and sunlight and eventually cause yield depression [2]. Palms that grow where there is *Imperata cylindrica* are generally stunted and retarded in growth [2]. Yeow et al. [3] reported a 20% yield reduction in oil palm plantation output caused by weeds. Soft grasses such as *Axonopus* sp., *Digitaria* sp., and *Palspalum* sp. have the ability to prevent weed succession of noxious species simply because base land for the noxious weeds to colonise is less available [4].

Among different noxious weed species, *Asystasia gangetica *is frequently found in oil palm plantations [5]. The eradication of very dense stands of *A. gangetica *in an oil palm plantation resulted in a 12% increase in fresh fruit bunch production [6]. *A. gangetica *spreads very quickly in most Malaysian plantations and small holdings. It adapts well to almost all types of soil, especially to well aerated deep soils, peat soils, and sandy beaches [7–9].

Weed control in oil palm plantations contributes to 75% of the total cost of pest management. The use of herbicides in crop protection is becoming a common practice worldwide, and it was estimated that in 2007, 72% of chemicals used in agriculture in Malaysia were herbicides [10].

In tropical Asia, legume cover crops are frequently planted in oil palm plantations to provide ground cover after forest clearing [11]. The wide spacing of palms at planting exposes the newly-uncovered soil to intense rainfall resulting in soil erosion and nutrient and organic matter loss [12]. Ultimately, the legume cover crops become shaded out, and soft grasses such as *Axonopus compressus*, *Cytococcum *sp., and *Paspalum conjugatum *and light ferns cover the field. Finally, noxious weeds like *Asystasia* and *Mikania *can dominate in these areas because of their high tolerance to low soil fertility and shade from the palm canopy [13]. In 10-year-old palms on a coastal soil in Malaysia, 15% of light reached the ground [12].

*A*. *compressus* is one of the soft grass species that is widely used as ground cover to protect soil erosion, as turf grass for landscaping and for sports fields as well as to conserve soil moisture in Malaysia [14]. Rika et al. [15] found that the coconut yield was highest when *A. compressus* was used as ground cover under coconut plantations compared with other grass species used as ground cover. This grass has a high potential for use as a cover crop to suppress weeds in plantations, especially areas that are dominated by broadleaf weeds and where establishing legume cover crops is not feasible. Soft grasses like *A. compressus* are also better at collecting loose fruits than broadleaf legume cover crops. Therefore, we hypothesized that *A. compressus *might control weeds under oil palm canopy in Malaysia.

Replacement series designs are frequently used to characterize the competitive interactions of species in mixed stands [16, 17]. Under this approach, species are grown in a fixed density, varying their proportions [18] to determine which species is the strongest competitor based on variables calculated from the replacement series data. Relative yield and total relative yield are variables that are frequently used to infer competitiveness between species [19]. By using multiple densities, it is possible to compare monoculture stands, allowing determination of the relative extent of intra- and interspecific competition between the species [20]. 

Despite the fact that *Axonopus compressus* is a dominant soft grass weed in oil palm plantations, there is no direct comparative study of this weeds with oil palm weeds. Therefore, the objectives of the study were to examine the interference dynamics between *Axonopus compressus* and the *Asystasia gangetica,* in different light conditions.

## 2. Materials and Methods

### 2.1. Experimental Site

Two separate experiments were conducted during January to March 2011 at Universiti Putra Malaysia (UPM), Malaysia (3° 02′ N, 101° 42′ E; elevation 31 m). The local climate was hot, humid, and tropical with abundant rainfall throughout the year. During the experimental period, monthly average maximum and minimum temperatures and relative humidity ranged from 33.5 to 34°C, 23 to 23.3°C, and 93.4 to 96%, respectively, while sunshine hours ranged from 6.31 to 7.06 hr d^−1^. Planting medium was prepared by mixing top soil, sand, and peat moss in a ratio 8 : 2 : 1 (v/v).

### 2.2. Plant Materials

*Axonopus compressus *cuttings consisting of two nodes 4 cm long were collected from the Plants House of UPM. *Asystasia gangetica *seeds were collected from an oil palm field in UPM and stored at room temperature for 3 months prior to seeding.

### 2.3. Experimental Design and Treatments

**Figure 1:**
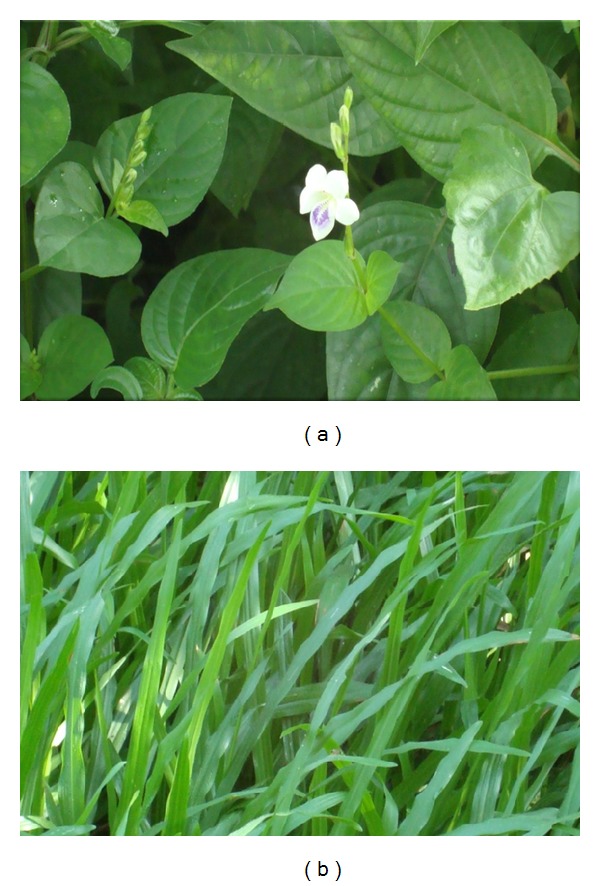
*Asystasi gangetica* (a) and *Axonopus compressus* (b).

The competitive potential of *Axonopus compressus* and *Asystasia gangetica *(Figure 1) was studied using multiple-density multiple-proportion replacements series. One experiment was conducted in a glasshouse under shade such that the rate of penetration of light was 40% and another one was conducted in full sunlight. Before starting study, photosynthetically active radiation (PAR) was measured using an illuminometer (Extech instruments, model 407026) on the soil surface of poly bags at full sunlight and glasshouse under shade. The percentage of PAR penetrating was then calculated. Both the experiments were carried out in a randomized complete block design with four replications. Population densities used were 72 and 288 plants m^−2^ with five *A. compressus *(C) to *A. gangetica *(W) proportions (C_100_ : W_0_, C_75_ : W_25_, C_50_ : W_50_, C_25_ : W_75_ and C_0_ : W_100_). The densities of 72 and 288 plants m^−2^ were considered appropriate based on the findings of previous work [21]. Polythene grow bags (poly bags) measuring 30 cm × 20 cm × 25 cm were used for growing the plants.

### 2.4. Plant Establishment

Seeds of *A. gangetica *were directly planted in poly bags filled with planting media for the given density, and thinning was done one week after emergence. At the same time *A. compressus *cuttings were planted in poly bags according to the required spatial arrangement. After planting, each poly bag was treated with 0.2% benomyl. *A. compressus *was allowed to interact for 10 weeks after planting with *A. gangetica*. Plants were maintained under nonlimiting water and nutrient conditions by providing fertilizer and watering. Other weeds were removed during the experimental period.

### 2.5. Data Collection

Plant heights from the soil surface to the top of the cover crops canopy were measured. Leaves of cover crops and weeds were harvested from each individual poly bag, and the leaf area of each species was determined using a leaf area meter (LI-3100, USA). Stems and leaves were placed by species into paper bags and dried at 72°C for 3 days to obtain shoot biomass. Cover crop and weed species shoot biomass data were converted to relative yield (RY) according to the following equations:

(1) $$\begin{array}{c} {\text{RY}}_{\text{crop}\;\text{species}}=\frac{\text{yield}\;\text{of}\;\text{weed}\;\text{species}\;\text{in}\;\text{mixture}}{\text{yield}\;\text{of}\;\text{weed}\;\text{species}\;\text{in}\;\text{monoculture}},\\ {\text{RY}}_{\text{weed}\;\text{species}}=\frac{\text{yield}\;\text{of}\;\text{weed}\;\text{species}\;\text{in}\;\text{mixture}}{\text{yield}\;\text{of}\;\text{weed}\;\text{species}\;\text{in}\;\text{monoculture}}. \end{array}$$

Regression of RYs on weed proportions 0, 0.25, 0.5, 0.75, and 1.0 were used to produce the replacement series diagrams to determine the competitiveness in the mixture as compared with the monoculture [18, 22, 23]. The shape of the replacement curve of the RY for the shoot dry weight relative to expected yields was used as the indicator of the extent of interference between the two competing species [24].

Relative yield totals (RYTs), which predict the competition between the two species for the same resources, were calculated as described by Santos et al. [25] by using the following equation:

(2) $$\begin{array}{c} \text{RYT}=\;{\text{RY}}_{\text{crop}\;\text{species}}+{\text{RY}}_{\text{weed}\;\text{species}}. \end{array}$$

The relative crowding coefficient (RCC), which serves as an index of competition when two species are mixed in equal proportions, was determined using the following equation [20]:

(3) $$\begin{array}{c} \text{RCC}=\frac{W1m/W2m}{W1p/W2p}\;\text{or}\;\frac{W2m/W1m}{W2p/W1p}, \end{array}$$

where *W*1*m* and *W*2*m* are shoot dry weight per pot of crop species and weed species at C_50_ : W_50_ mixture and *W*1*p*, and *W*2*p* are shoot dry weight per pot of crop species and weed species in pure culture (monoculture). Equivalent yield ratios (EYR) or the proportion at which both species growing in the mixture produce the same yield was calculated for each mixture [25].

### 2.6. Statistical Analysis

Plant height and leaf area data were analysed by the analysis variance using SAS statistical software package version 9.2 [26], and values were further differentiated by Tukey's test at *P* ≤ 0.05. All regressions were conducted using Sigma Plot version 11.

## 3. Results

### 3.1. Competitive Ability of *A. compressus* against *A. gangetica* in Full Sunlight

Since density by proportion interaction was significant for shoot dry weight of *A. compressus*, every combination was analyzed separately (data not given). *A. compressus* in monoculture (C_100_), C_75_ : W_25_ and C_50_ : W_50_ produced less shoot biomass per plant than in C_25_ : W_75_ at two different densities (Table 1). Moreover, the highest shoot dry weights plant^−1^ across the different proportions was found at 288 plants m^−2^. The mean shoot dry weight per plant of *A. gangetica* decreased as the proportions of *A. compressus* increased. The highest *A. gangetica* shoot dry weight was obtained in the pure stand of *A. gangetica* (W_100_) and the lowest in C_75_W_25_ and C_50_W_50_ at 288 plants m^−2^. At 72 plants m^−2^ density, the lowest *A. gangetica* shoot dry weight was in C_75_W_25_ (Table 1), and other proportions did not show any significant difference from the pure stand. 

Plant height of *A. compressus* was unaffected in different proportions. *A. gangetica *had the highest plant height in monoculture and a reduced height in mixtures. In General, plant heights of *A. compressus* were lower than *A. gangetica* in monoculture (Table 1). Leaf areas of *A. compressus* in association with *A. gangetica* showed a similar trend to the shoot dry weight at 288 plants m^−2^ density as *A. compressus* had the highest leaf area in C_25_W_75_. Although *A. compressus* showed this response at 72 plants m^−2^ density, it was not significant (Table 1). *A. gangetica* leaf area decreased with increasing *A. compressus* proportions in the mixture as *A. gangetica *had the lowest leaf area in C_75_W_25_ at 72 plants m^−2^ and in C_75_W_25_ and C_50_W_50_ at 288 plants m^−2^ (Table 1).

**Figure 2:**
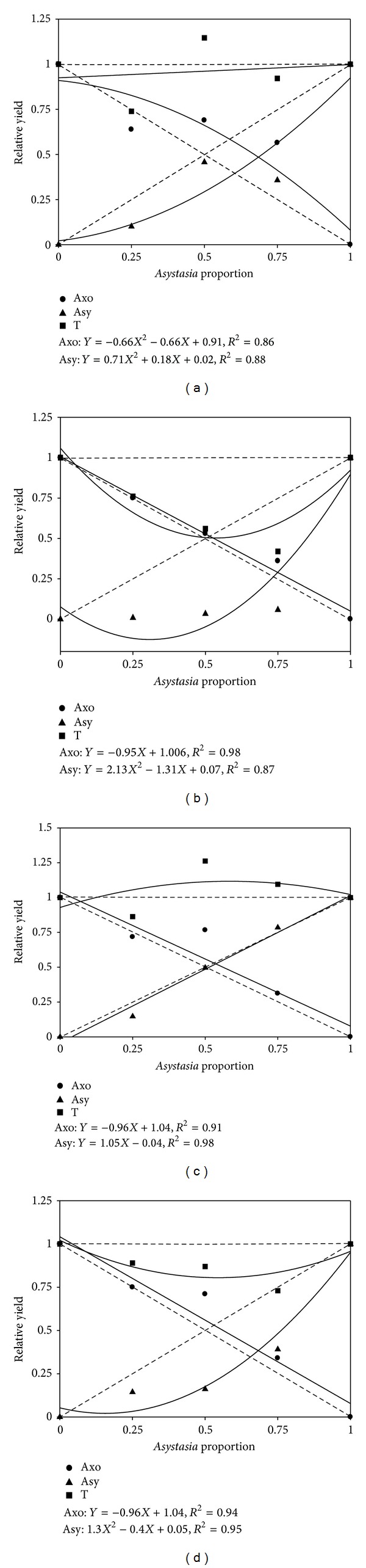
Interaction between *Asystasi gangetica* (Asy) and *Axonopus compressus* (Axo) in full sunlight with low density (a), *A. compressus* in full sunlight with high density (b), *A. compressus* in shade with low density (c), and *A. compressus* in shade with high density (d) in replacement series experiments at 10 wk after planting. T = total.

The De Wit competitiveness diagrams of the relative shoot dry weight (RY) of the *A. compressus* and *A. gangetica* is shown in Figures 2(a) and 2(b). The RY of *A. compressus* (Figure 2(a)) increased in a quadratic manner as the proportion of it in mixtures with *A. gangetica* increased, resulting in a convex curve at 72 plants m^−2^. As the proportion of *A. gangetica *in the mixtures increased, the RY of *A. compressus* decreased in a linear manner and near to the expected curve at 288 plants m^−2^ (Figure 2(b)). *A. gangetica *responded to *A. compressus* to form a concave curve at both densities (Figures 2(a) and 2(b)) that resulted in an equivalent yield ratio (EYR) of more than 0.50. The EYRs were 0.68 and 0.75 for *A. compressus* at 72 and 288 plants m^−2^, respectively (Figures 2(a) and 2(b)). The RYT of mixture was equal to monoculture at 72 plants m^−2^ (Figure 2(a)). The RYT value was less than 1 at 280 plants m^−2^ (Figure 2(b)). The Relative crowding coefficient (RCC) values of *A. compressus* at both densities, when grown in equal proportions, were more than the RCCs of *A. gangetica* (Table 3). 

### 3.2. Competitive Ability of *A. compressus* against *A. gangetica* in Shade (Glasshouse Condition)

Shoot biomass of *A. compressus* at 72 plants m^−2^ did not show a significant difference amongst the different proportions, but at 288 plants m^−2^ density the *A. compressus* in C_25_W_75_ and C_50_W_50_ produced more shoot biomass per plant than in monoculture (C_100_) and in C_75_W_25_ (Table 2). *A. gangetica *produced the highest shoot biomass per plant in C_25_W_75_ proportion in both densities. However, *A. gangetica *produced the lowest shoot biomass in C_75_W_25_ at 72 plants m^−2^ and in C_75_W_25_ and C_50_W_50_ at 288 plants m^−2^ (Table 2).

With the increasing proportion of *A. gangetica *in mixtures, plant height of *A. compressus* decreased at both densities (Table 2). Plant height of *A. gangetica *was unaffected at 72 plants m^−2^ but decreased with increasing proportion of *A. compressus *in mixtures at 288 plants m^−2^. *A. compressus* leaf area did not show any difference between the different proportions at 72 plants m^−2^ and had the biggest leaf area in C_25_W_75_ and C_50_W_50_, at 288 plants m^−2^ (Table 2). Leaf area of *A. gangetica* decreased with increasing *A. compressus* proportions in the mixture as *A. gangetica *had the lowest leaf area in C_75_W_25_ at both densities. *A. gangetica* had the highest leaf area in C_25_W_75_ at both densities compared with monoculture at both densities (Table 2). 

Replacement series curves of interaction of *A. gangetica* with *A. compressus* in the shade have been illustrated in Figures 2(c) and 2(d). The RY of *A. compressus* increased in a linear manner and more than expected as the proportion of it in mixtures with *A. gangetica* increased at both densities. The response of *A. gangetica* to *A. compressus* with normal density under the shade was linear and resulted in a 0.56 EYR (Figure 2(c)). *A. gangetica* responded to *A. compressus* to form a concave curve at 288 plants m^−2^ density (Figure 2(d)) that resulted in an EYR of about 0.68. The RYT value at 72 plants m^−2^ was greater than 1.0 (Figure 2(c)). The RYT value was less than 1.0 at 280 plants m^−2^ (Figure 2(d)). The RCC values of *A. compressus* in both densities, when grown in equal proportions, were more than RCCs of *A. gangetica* on them (Table 3).

## 4. Discussion 

*A. compressus* shoot dry weight increased despite a decreasing number of *A. compressus* when grown with *A. gangetica* compared to monoculture at 72 and 288 plants m^−2^ in the open and at 288 plants m^−2^ in the shad. It appears that *A. compressus* produces more dry weight per plant when competing against *A. gangetica* than in monoculture. *A. compressus* shoot dry weight remained unchanged from C_100_W_0_ to C_50_W_50_, hence, as proportions changed in this range both intra- and interspecific competition were counteracting each other. However, as fewer *A. compressus* plants were in the mixture (C_25_W_75_), neighbouring plants apparently did not compete with each other, resulting in no intraspecific competition. Under these conditions, *A. compressus* was more efficient in competing against *A. gangetica* than against other *A. compressus* plants. By contrast, shoot dry weight of *A. gangetica* decreased with increasing *A. compressus* proportions in the mixture as *A. gangetica* had the lowest shoot dry weight in C_75_W_25_ at both densities in the open and shade. These findings suggest that *A. compressus* responded plastically to competition, whereas *A. gangetica* did not. The greater biomass of *A. compressus *compared with *A. gangetica* in a mixture would result in a greater demand for resources. Other studies have also shown that more competitive species produce a higher relative yield when grown in mixtures, whereas the yield of weak competitors is lower in mixtures than in monoculture [27, 28]. When one plant of basil (*Ocimum sanctum*) was competing with three of weed species, plant height and fresh weight plant^−1^ of basil increased [29]. Overyielding has been associated with higher biomass density and light interception or greater demand for resources [28, 30, 31]. 

*A. gangetica* at both densities in the open grew better in monoculture compared to C : W mixtures, indicating *Asystasia gangetica* was more affected by interspecific interactions. By contrast,* A. gangetica *at both densities in the shade in C_25_W_75_ proportion had more shoot dry weight relative to monoculture. It seems that *A. gangetica* plants in the shade in C_25_W_75_ did not compete with each other in this proportion and were more competitive against *A. compressus* which helped to neutralize additional interspecific completion by *A. compressus* in this proportion. Therefore, *A. gangetica* grew better in the mixture than in monoculture and was a better competitor than the *A. compressus* in the shade compared to the open. Moreover, *A. compressus* did not over yield at 72 plants m^−2^ in the shade at C_25_W_7_. It appears that *A. compressus* was less efficient in competing against the *A. gangetica* in low density.

Plant height of *A. gangetica* was reduced in different proportions in the open. Generally, plant heights of *A. compressus* were lower than *A. gangetica* in monoculture in the open, but unaffected by competition. Plant height of *A. compressus* decreased at both densities in the shade. Plant height of *A. gangetica* decreased with increasing proportion of *A. compressus* in mixtures at 288 plants m^−2^ but was unaffected at 72 plants m^−2^ in the shade. Variation between plant heights of *A. compressus* and *A. gangetica* in the shade was lower than in the full sunlight, because in the shade they were trying to achieve more light for growth. *A. compressus* and *A. gangetica* are shade tolerant [9, 14]. Shading has been shown to drastically reduce plant growth [32]. Despite this fact, *A. gangetica* canopy height in the open, which was taller than *A. compressus*, did not influence *A. compressus* height, but *A. gangetica* canopy shade could effect on height of *A. compressus* and reduce that in both densities in the shade.

Leaf areas of *A. compressus* in association with *A. gangetica* also responded in a similar way to shoot dry weight at 72 and 288 plants m^−2^ in the open and at 288 plants m^−2^ density in the shade. Thus, *A. compressus* had the biggest leaf area in C_25_W_75_. Greater leaf area expansion rates would favour *A. compressus* plants in competition for light. By contrast, leaf area of *A. gangetica* decreased with increasing *A. compressus* proportions in the mixture as *A. gangetica *had the lowest leaf area in C_75_W_25_ at both densities in the open and shade. Plant size suggests a potential advantage for light capture and greater penetration of PAR to the soil surface [33].

As the proportion of *A. gangetica* in the mixtures increased in open, *A. compressus* yielded over the expected rate and produced a convex curve at 72 plants m^−2^ and a linear response with more than expected at 288 plants m^−2^, whereas *A. gangetica* yielded under the expected rate and had a concave curve at both densities in the open. Response of *A. gangetica* to *A. compressus* with normal density (72 plants m^−2^) under the shade was linear and less than expected, and *Asystasia gangetica* responded at 288 plants m^−2^ density to *A. compressus* under shade to form a concave curve. A convex curve for one species and a concave curve for the other species in the series indicate that the species are competing for a common resource. When both curves are convex and concave, mutually stimulatory and antagonistic relations are indicated, respectively [24]. The RY of *A. compressus* increased in a quadratic manner or linearly to more than expected, while RY of *A. gangetica* in interaction with *A. compressus* was concave or linear but less than expected in all conditions, indicating that *A. gangetica* was more affected by interspecific interactions with *A. compressus* and was less competitive than *A. compressus*. 

If the RY curves intersect at 50 : 50 proportions, the two competing species are relatively equal in competitiveness [25]. The RY of *A. gangetica* increased in a linear or nonlinear manner as its proportion in the mixture with *A. compressus* increased, but its RY was not equivalent to that of *A. compressus* when each comprised half the mixture. This resulted in EYR to equal to 0.75 at 288 plants m^−2^ in the open. This meant that about one *A. compressus* plant equaled the shoot dry weight production of three *A. gangetica *plants compared to the total shoot dry weight production of each monoculture, and suggesting that a large population of *A. gangetica* is needed to suppress a smaller population of *A. compressus*. The EYR for *A. compressus* at 72 in the open was 0.68. Response of *A. gangetica* to *A. compressus* at 72 and 288 plants m^−2^ in the shade resulted in a 0.56 and 0.68 EYR, respectively. *Axonopus compressus *seemed to be more competitive than *A. gangetica *at 288 plants m^−2^ in the open compared to other conditions, and *A. compressus* was not a good competitor at 72 plants m^−2^ in the shade. For EYR, the *A. compressus* performed in the following order: *A. compressus* at 288 plants m^−2^ in the open > *A. compressus* at 72 plants m^−2^ in the open and *A. compressus* at 288 plants m^−2^ in the shade > *A. compressus* at 72 in the shade.

An RYT value around 1 indicates that the same resource or area is being used by the two competing species (overlap in resource utilization) [20]. An RYT > 1 indicates some niche differentiation between the species, where competition is either avoided or minimized [17]. However, other processes can also produce RYT > 1, indicating facilitation where one species benefits another [34]. An RYT < 1 suggests mutual antagonism. The RYT value was less than 1 at 280 plants m^−2^ in the open and shade, and 72 plants m^−2^ in the open means that mutual antagonism is occurring with the species producing less than expected when grown together [35]. Anyway, RYT reduced in the following order: 280 plants m^−2^ in the open > 280 plants m^−2^ in the shade > 72 plants m^−2^ in the open. It is likely allelopathy to be involved in the interaction of *A. compressus* and *A. gangetica* at 280 plants m^−2^ in the open and shade, because the RYT value of *A. compressus* with *A. gangetica* was < 1 for all proportions. The occurrence of allelopathic interaction would have lowered the total yield in mixtures compared with monoculture [16]. The RYT value at 72 plants m^−2^ in the shade was greater than 1.0 suggesting that the two species made different demands on resources leading to better growth of *A. compressus* or that this crop mixture was less affected by interspecific competition than by intraspecific competition, facilitating over yielding (RYT >1). 

The relative crowding coefficient (RCC) value demonstrates the aggressiveness of one species towards another. The greater (RCC) values of *A. compressus* than the RCCs of *A. gangetica* at both densities in the open and shade, when grown in equal proportions, confirms the aggressiveness of *A. compressus* against the *A. gangetica* in terms of shoot dry weight production. When competing for limited resources, the species with the greater RCC in the mixture is the strongest competitor [17]. 

The superior competitiveness of *A. compressus* relative to *A. gangetica* is likely resource competition. *Axonopus compressus* became extremely dense fast, thereby limiting the space available to the weed population and suppressing *A. gangetica* growth. Fast growth result in a superior plant [36]. Furthermore, plant size suggests a potential advantage for light capture and greater penetration of PAR to the soil surface, making the crop less competitive against weeds [33]. Also, rapid growth by lateral spread of *A. compressus* through tillering seemed to be the reason for superiority of *A. compressus* in competition with *A. gangetica*. Greater tiller production is one of the factors associated with superior suppression [37]. The aggressiveness of *A. compressus* can also be explained in terms of its prolific rooting system, which enabled it to capture more of the limited soil water and nutrients and resulted in rapid growth in terms of biomass accumulation and canopy development [38]. Moreover, the presence of allelopathic interaction would have lowered the total yield in mixtures compared with the monocultures [16]. *Axonopus compressus* is known to produce allelochemicals that affect the growth of other plants [39]. There are some reports that demonstrate *A. compressus* had competitive ability. Oka Nurjaya [21] reported that in mixtures, *A. compressus* was more competitive than grass and legume species. Rika et al. [15] observed that under coconut, a local cultivar of *A. compressus* produced higher yields than other grasses. *Axonopus* sp., *Digitaria* sp., and *Palspalum* sp. are classified as soft weeds in oil palm plantation which maintain the balance of the weed flora and prevent weed succession by noxious species simply because the base land for the noxious weeds to colonise is less available [4, 40]. On most oil palm plantations in the far East, the cover that establishes itself is a mixture of the fern *Nephrolepis biserrata* with varying components of grasses such *Paspalum conjugatum* and *Axonopus compressus* [41, 42].

Based on the present findings, it can be concluded that *A. compressus *is highly competitive against *A. gangetica*. However, *A. compressus* under shade uses most of its energy to achieve more light for growth, and hence, *Axonopus compressus* density should be higher under shade to increase its competitiveness with *A. gangetica*. Further research into the *A. compressus* competitive ability with weeds is planned.

## Figures and Tables

**Table 1:** Shoot dry weight, plant height, and leaf area of *A. compressus *and *A. gangetica* in monoculture and mixtures at 10 wk after planting in full sunlight.

Treatments	Shoot dry weight plant^−1^ (g)		Plant height (cm)		Leaf area plant^−1^ (cm^2^)	
	*A. compressus *	*A. gangetica *	*A. compressus *	*A. gangetica *	*A. compressus *	*A. gangetica *
72 plants m^−2^						
C_100_	3.1b	—	4.5a	—	244.6a	—
C_75_W_25_	2.8b	0.8b	5.5a	17.0b	238.7a	32.0b
C_50_W_50_	4.2b	1.9a	4.8a	20.0b	355.3a	123.3a
C_25_W_75_	6.6a	1.0ab	3.9a	15.0b	479.9a	243.3a
W_100_	—	2.1a	—	27.7a	—	142.8a
288 plants m^−2^						
C_100_	10.4b	—	5.0a	—	613.3b	—
C_75_W_25_	9.7b	0.02c	4.5a	7.0b	601.6b	2.3c
C_50_W_50_	10.4b	0.12c	4.5a	6.8b	636.0b	13.7c
C_25_W_75_	16.4a	0.48b	5.0a	6.2b	909.3a	40.5b
W_100_	—	1.99a	—	20.9a	—	134.3a

Means within column for each density followed by the same letter are not significantly different at *P* = 0.05%.

C: *A. compressus*, W: *A. gangetica*.

**Table 2:** Shoot dry weight, plant height, and leaf area of *A. compressus *and *A. gangetica* in monoculture and mixtures at 10 wk after planting in shade.

Treatments	Shoot dry weight plant^−1^ (g)		Plant height (cm)		Leaf area plant^−1^ (cm^2^)	
	*A. compressus *	*A. gangetica *	*A. compressus *	*A. gangetica *	*A. compressus *	*A. angetica *
72 plants m^−2^						
C_100_	3.3a	—	15.0a	—	201.9a	—
C_75_W_25_	3.4a	0.21c	15.0a	19.3a	204.6a	29.2c
C_50_W_50_	5.1a	1.07b	13.0c	21.8a	307.7a	221.4b
C_25_W_75_	4.1a	2.40a	14.0b	18.1a	254.0a	436.3a
W_100_	—	1.08b	—	23.9a	—	144.5b
288 plants m^−2^						
C_100_	7.4b	—	19.0a	—	477.3b	—
C_75_W_25_	6.8b	0.20c	19.0a	21.8a	499.2b	20.9d
C_50_W_50_	9.6a	0.33c	14.6b	19.0b	703.7a	47.0c
C_25_W_75_	9.3a	1.66a	14.6b	20.8ab	660.8a	163.5a
W_100_	—	1.03b	—	21.3ab	—	133.1b

Means within column for each density followed by the same letter are not significantly different at *P* = 0.05%.

C: *A. compressus*, W: *A. gangetica*.

**Table 3:** Relative crowding coefficient in interaction between *A. compressus *and *A. gangetica. *

Treatments	Relative crowding coefficient *A. compressus/A. gangetica *	Relative crowding coefficient *A. gangetica*/*A. compressus *
In full sunlight		
72 plants m^−2^	1.51	0.66
288 plants m^−2^	4.04	0.24
In shade		
72 plants m^−2^	1.01	0.90
288 plants m^−2^	1.54	0.64
